# Assessing the future medical cost burden for the European health systems under alternative exposure-to-risks scenarios

**DOI:** 10.1371/journal.pone.0238565

**Published:** 2020-09-11

**Authors:** Yevgeniy Goryakin, Sophie P. Thiébaut, Sébastien Cortaredona, M. Aliénor Lerouge, Michele Cecchini, Andrea B. Feigl, Bruno Ventelou

**Affiliations:** 1 OECD, Directorate for Employment, Labour and Social Affairs, Paris, France; 2 Imperial College Business School, South Kensington Campus, Kensington, London, United Kingdom; 3 Aix-Marseille University, CNRS, EHESS, Centrale Marseille, IRD, AMSE, Marseille, France; University of Genoa, ITALY

## Abstract

**Background:**

Ageing populations and rising prevalence of non-communicable diseases (NCDs) increasingly contribute to the growing cost burden facing European healthcare systems. Few studies have attempted to quantify the future magnitude of this burden at the European level, and none of them consider the impact of potential changes in risk factor trajectories on future health expenditures.

**Methods:**

The new microsimulation model forecasts the impact of behavioural and metabolic risk factors on NCDs, longevity and direct healthcare costs, and shows how changes in epidemiological trends can modify those impacts. Economic burden of NCDs is modelled under three scenarios based on assumed future risk factors trends: business as usual (BAU); best case and worst case predictions (BCP and WCP).

**Findings:**

The direct costs of NCDs in the EU 27 countries and the UK (in constant 2014 prices) will grow under all scenarios. Between 2014 and 2050, the overall healthcare spending is expected to increase by 0.8% annually under BAU. In the all the countries, 605 billion Euros can be saved by 2050 if BCP is realized compared to the BAU, while excess spending under the WCP is forecast to be around 350 billion. Interpretation: Although the savings realised under the BCP can be substantial, population ageing is a stronger driver of rising total healthcare expenditures in Europe compared to scenario-based changes in risk factor prevalence.

## Introduction

In Western Europe, NCDs account for about 91% of all deaths and 88% of all disability-adjusted life years (DALYs) [1]. They also strain healthcare budgets, with five major NCDs- cardiovascular and endocrine conditions, cancers, respiratory and mental disorders- contributing to over a third of total health spending in high income countries [2]. The societal impacts of NCDs will become more severe in decades to come, as population ageing and the rising prevalence of NCDs are expected to put mounting financial pressure on European healthcare systems and on the labor markets [3–5]. However, forecasting the future magnitude of this burden is difficult, due to demanding data requirements and the need to make strong assumptions about future trajectories of health expenditures and underlying risk factors. For example, since a large part of the NCD burden is preventable [6], future medical costs are likely to be driven by the evolution of underlying risk factors such as obesity, smoking and harmful alcohol consumption. In addition, future medical costs will depend on the evolving size and structure of the population and its aging process; pattern and volume of healthcare use, as well as changes in the medical technology and the associated costs.

Although various efforts to forecast medical costs have been made, both on country [7] and regional level [8], a general criticism of the existing forecasting methods is that they do not take into account major epidemiological changes [9], thus treating future risk factor exposure as fixed. In addition, a number of macro-level socioeconomic determinants such as regulatory role of government, income inequality, urbanization and globalization may have a strong influence on the future evolution of risk factors [10–12].

A distinguishing feature of our study is its consideration of the evolution of medical costs under various plausible scenarios on the distribution of risk-factors across the whole population of the 27 EU countries and the UK, while taking into account both demographic and socioeconomic future trends. Having said that, although we will provide results according to the central predictions of our model, generating precise health expenditure forecasts until 2050 is not our goal, as such efforts can become notoriously unreliable even under much shorter time horizons. Rather, we are more interested in potential monetary savings and losses under alternative scenario assumptions, where we consider how direct medical costs may evolve in the future given what we call best case prediction (BCP) and worst case prediction (WCP), compared to the BAU trajectory based on current socioeconomic trends.

This analysis formed part of the FRESHER H2020 consortium, funded by the European Union H2020 program, linking several institutions in Europe and gathering the evidence from relevant disciplines in the domain of healthcare need projections, including public health and epidemiology, foresight sciences, economics and sociology. As a result, a dynamic microsimulation model designed to estimate the impacts of behavioral risk factors on chronic diseases and their direct economic costs in the EU 27 countries and the UK as well as in the sub-regions, was developed. This model also fills the gap in the existing tools in that it combines the benefits of microeconomic analyses with their focus on individual behaviors, with macroeconomic analyses more concerned about future projection of aggregate trends.

## Methods

### Microsimulation model design

The microsimulation model was developed to forecast future chronic disease burden, longevity and direct economic costs in 27 EU countries and the UK until the year 2050, as well as the extent to which future changes in epidemiological trends and specific policies can modify these outcomes. This modelling platform, initially based on previous OECD work [13], was further improved as part of the FRESHER project [14]. The model uses case-based microsimulation to create representative synthetic life histories from birth to death, and relies on detailed epidemiological and demographic information from various sources. The model also uses prevalence-based direct cost estimates as an input into the model to forecast incidence-based health expenditures associated with various scenarios/policy interventions, from the health system perspective. More detailed information on data and the modelling assumptions can be found at a technical report posted online [14,15], the accompanying paper [16], as well as in the S6 Appendix in S1 File.

### Diseases modelled

In general, attempt has always been made to estimate the direct medical costs for the following NCDs, although in some countries we evaluated costs only for a subset of these diseases due to the limited data (for more information, see S1 Appendix in S1 File):

- Ischemic stroke
- Haemorrhagic stroke
- Myocardial infarction and chronic heart disease
- Cancers
- Diabetes
- Chronic kidney disease (CKD)
- Chronic Obstructive Pulmonary Disease (COPD)
- Cirrhosis
- Depression
- Neurologic disorders (including Alzheimer disease and dementia)
- Injuries (including intentional and unintentional injuries and self-harm)

We selected these diseases due to their large contribution to the total NCD burden, as well as because many of them have common aetiologies and potentially modifiable risk factors such as obesity and harmful alcohol consumption [17,18], and therefore are likely to be amenable to the scenario assumptions. In addition, since injuries (including intentional, unintentional and self-harm) are significantly linked with alcohol use disorders [19], representing about 10% of the total disease burden caused by the alcohol use [20], the cost of injuries was modelled as well. Additional information on how the injury costs were estimated is provided in S2 Appendix in S1 File.

### Estimation strategy

The microsimulation model requires individual-level total medical costs, conditional on age, gender and disease status as an input. In a standard bottom-up approach to cost estimation, patient-level units of health care attributable to a specific disease are multiplied by a price for this unit, and then summed up. However, in practice administrative coding is often not very informative about the underlying reason for incurring health expenditures. For example, falls can occur excessively in people suffering from Alzheimer’s disease, but health expenditures may be recorded under alternative coding, thus giving an incomplete picture of the health expenditure burden facing this group. Alternatively (and also incorrectly), all the recorded costs for a person with a disease can be attributed to this disease. To overcome this limitation, we estimated costs using regressions, as the mean marginal difference of the predicted medical costs with a disease variable switched on or off (i.e., using between subjects comparison). This approach is commonly used to estimate incremental costs for select diseases and risk factors [21–23]. In our study, the outcome variable is the total cost of hospital and ambulatory care (including costs of drugs) at the patient level.

We also account for the possibility that having two or more diseases may lead to costs that are greater than a simple sum of costs for separate diseases [24]. For more information about how this super-additivity was modelled, see the S1 Appendix in S1 File.

Finally, we estimated age- and gender-specific residual medical costs for people who had no model- defined diagnosed diseases (but could have other diagnosed conditions, or were otherwise healthy). Taking into account such costs is important because reducing risk factor prevalence may still lead to accruing costs for unrelated medical conditions, either because people will live longer, or because they can be affected by unrelated competing disease risks. For more details about the estimation approach, see the S1 Appendix in S1 File.

### Data sources and cost extrapolation

Although average healthcare spending per capita was accessible for all European countries, precise cost-of-illness estimates were not available, or even calculable, for a great majority of countries. A cost extrapolation model was therefore used to generalize the incremental costs of our 10 chronic conditions from the three “anchor countries” where the data was available -France, Estonia and the Netherlands- to the remaining EU-28 countries.

Our extrapolation methodology assumes that the annual treatment cost differentials between countries are time-invariant, and mostly driven by the differences in two components: costs per unit of treatment received, as well as population-level intensity of the treatments provided. To estimate these differentials, we used the OECD data [25] on the inpatient curative and rehabilitative care spending per capita, outpatient curative and rehabilitative care spending per capita as well as medical goods spending per capita. To ensure comparison and to smooth over temporary data variability, we used this data averaged over 3 years: 2012, 2013 and 2014, expressed in current prices.

Given a significant amount of cost-based extrapolations and uncertainties associated with future modelled scenarios, it was decided to present results not separately for each country, but aggregated over three regions: Southern Europe, Central/Eastern Europe and Northern Europe. There is no consensus on how such a grouping should be done, therefore members of the Fresher consortium decided to follow simple geographical criteria at the beginning of the project. First, Southern Europe is composed of EU27 countries bordering the Mediterranean Sea, as well as Portugal. Central/Eastern Europe counties include those which joined the EU since 2004, except Slovenia and Croatia (which belong in the Southern Europe group). The remaining countries belong in Northern Europe group. The final groupings were as follows:

1. Southern Europe (France, Italy, Spain, Portugal, Cyprus, Greece, Croatia, Malta, Slovenia);
2. Central/Eastern Europe (Estonia, Bulgaria, Poland, Romania, Slovakia, Hungary, Latvia and Lithuania);
3. Northern Europe (the Netherlands, Austria, Belgium, Czech Republic, Denmark, Finland, Germany, Ireland, Luxembourg, Sweden and United Kingdom).

This setup is not supposed to indicate that countries belonging to one group are always more similar to each other than to countries in another group. For example, healthcare system in France can have much more in common with Belgium than with Croatia. Nevertheless, deciding on group composition is likely to involve at least some degree of arbitrariness, and we tried to minimize it by using simple geographical proximity criteria. In any case, grouping decisions are not likely to affect the quality of the extrapolation so long as the differentials in costs between countries are taken into account, as we describe in the S1 Appendix in S1 File.

### Disease identification

In source databases, we had to identify the occurrence of NCDs through a medical diagnosis and/or an associated treatment. Disease definitions were standardized across countries, with disease identification based on criteria such as International Classification of Diseases (ICD) diagnostic codes [26] and/or codes from the drug prescription databases. Specifically, in France, disease identification was done using ICD—version 10 codes; hospital discharge data as well as drug discharge Anatomical Therapeutic Chemical (ATC) codes, using Huber’s classification method [27]. In Estonia, identification was done using ICD-10 codes provided in the Estonian Health Insurance Fund (EHIF) dataset. In the Netherlands, identification of NCDs was only possible using ATC codes of medications prescribed to patients outside of hospitals recorded, which precluded reliable identification of diseases, such as CKD and cirrhosis. Therefore, for the Netherlands, we borrowed these later costs from France (only those missing). For more details about country-specific diseases identification, see the S1 Appendix in S1 File.

### Model scenarios

We compare the outcomes under three scenarios for the evolution of the following key risk factors: smoking; obesity rates; alcohol use; physical inactivity and high blood pressure. Our BAU scenario is identical with the “the rich get healthier” scenario described in more detail in the accompanying paper [16]. Briefly, a group of 90 health experts were asked to predict the future evolution of five key risk factors based on the narrative description of four different scenarios of the future, previously identified after consulting a large number of experts on societal trends likely to impact the future evolution of NCDs. The “the rich get healthier” projection, in which it was assumed that the societal inequalities will continue to widen, and mostly market-based solutions to societal and environmental problems will be offered, was chosen as the BAU, as it was viewed as the most likely to represent the future based on the continuation of the current trends.

To implement the best (worst) case scenarios, we first calculated the spread between the Europe-wide average rate for a given risk factor in 2015, and the lowest (highest) rate for that risk factor. For example, if the average obesity rate in Europe in 2015 was 20%, and the highest observed obesity rate that year was 30%, then we assume that every country will see its obesity rate linearly increase so that in 2050 it’s greater by 10 percentage points than its current rate. This approach was implemented to ensure comparability with the other scenarios described in the accompanying paper [16].

Table 1 presents the average risk factor prevalence in 2015, as well as their evolution under the three scenarios. See S3 Appendix in S1 File for a graphical depiction of the changes in risk factor prevalence over time.

**Table 1:** Risk factor prevalence (%) in 2015 (actual) and in 2050 (predicted).

Region	Risk factor	BAU, 2015	BCP, 2050	BAU, 2050	WCP, 2050
	Inactive	28.4	12	35.1	54.5
	Overweight	62	59.9	65.9	64.2
EU	HBP	27.9	24.5	35	44.6
	Smoker	22	10	17.8	31.2
	Hazard drinking	12.1	9	12.3	12.6
	Inactive	20.6	6.7	27.8	47.1
	Overweight	59.6	58.5	64.4	62.7
EE	HBP	35.1	33.3	43.7	53.1
	Smoker	24.4	12.3	20	33.1
	Hazard drinking	9.8	8.7	9.5	9.5
	Inactive	27	10.1	33.2	52.5
	Overweight	61.7	59.6	65.6	63.9
NE	HBP	24.9	20.6	31.2	40.8
	Smoker	20.6	9	17.1	30.4
	Hazard drinking	13.2	9.3	13.5	14
	Inactive	33.5	17	40.2	59.6
	Overweight	63.1	60.8	66.7	65
SE	HBP	28.3	25.2	35.8	45.4
	Smoker	22.4	10.2	17.8	31.1
	Hazard drinking	11.5	7.4	11.8	12.3

EE means Central and Eastern Europe; EU: European Union; NE is Northern Europe and SE is Southern Europe

**Fig 1:**
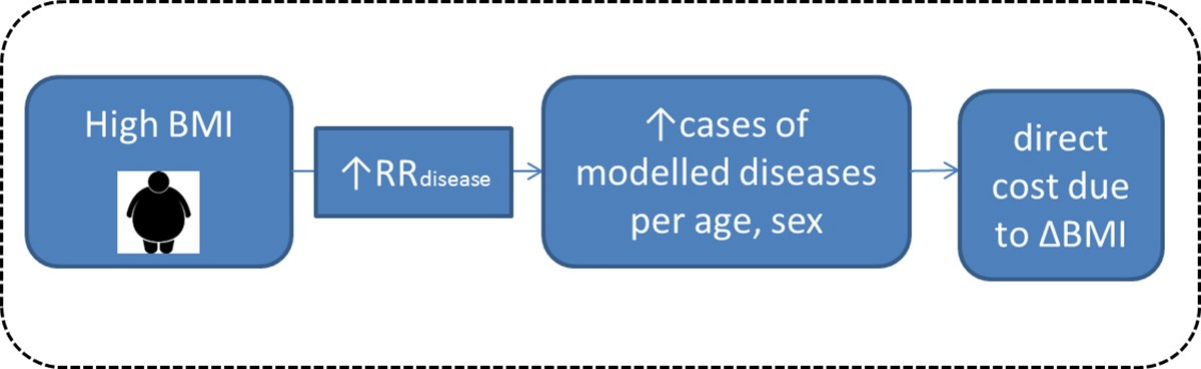
Conceptual framework for high BMI attributable direct costs.

Fig 1 depicts an example of a model-implemented link between an increase in one risk factor-the BMI- and an increased risk of developing related conditions such as heart disease or diabetes. Each additional disease arising out of an increased BMI is then associated with the disease-specific cost, or in the case of multiple conditions, with the cost for multiple diseases, which have been estimated for all 28 countries. A total cost saving attributable to the BCP can likewise be estimated.

## Results

All the costs are expressed in constant, 2014 prices. Between 2014 and 2050, the overall healthcare spending is forecast to grow at an annual rate of about 0·8% in the BAU case, from 966 billion Euros in 2014 to 1258 billion Euros in 2050 (Table 2).Total healthcare spending under the BCP is predicted to be lower than under BAU by 2.73% in 2050, while spending under the WCP is predicted to be higher by about 1.62%. Another way to look at this is that under BAU scenario, medical spending in the EU-27 countries and the UK will be greater in 2050 by 291 billion Euros than in 2014. On the other hand, WCP will lead to 312 billion greater spending in 2050 compared to 2014, which means that it only contributes about 7% more spending on top of the BAU scenario over this period. Likewise, BCP will only lead to 12% lower spending compared to the BAU scenario over this period.

**Table 2:** Evolution of total annual predicted costs (bn Euros) by region, year and scenario.

Region	Year	WCP	BAU	BCP	Excess spending*	Cost Savings**
	2014	966	966	966	0.00%	0.00%
EU	2030	1097	1089	1075	0.72%	1.29%
	2050	1278	1258	1223	1.62%	2.73%
	2014	36	36	36	0.00%	0.00%
EE	2030	37	37	37	0.53%	0.90%
	2050	38	37	37	0.80%	0.98%
	2014	547	547	547	0.00%	0.00%
NE	2030	627	622	612	0.81%	1.56%
	2050	743	728	701	1.98%	3.76%
	2014	366	366	366	0.00%	0.00%
SE	2030	423	421	417	0.57%	0.95%
	2050	498	493	485	1.02%	1.65%

*WCP vs BAU

**BCP vs BAU. Excess spending is presented as % of cost difference under WCP and BAU scenarios, relative to BAU; cost savings are difference between costs under BCP and BAU scenarios, relative to baseline. EE means Central and Eastern Europe; EU: European Union; NE is Northern Europe and SE is Southern Europe.

This narrow range of variation between BCP and WCP, each compared to BAU, was a first striking result. It indicates that population ageing is the major driver of the overall healthcare spending in Europe, with a relatively weak margin attributable to healthy behaviour improvements. By region, in 2050, the largest reductions in the healthcare budget under the BCP compared to BAU are predicted to occur Northern Europe (3.76%); followed by Southern Europe (1.65%) and Eastern Europe (0.98%). The same regional ranking will hold when considering excess spending under WCP scenario.

**Fig 2:**
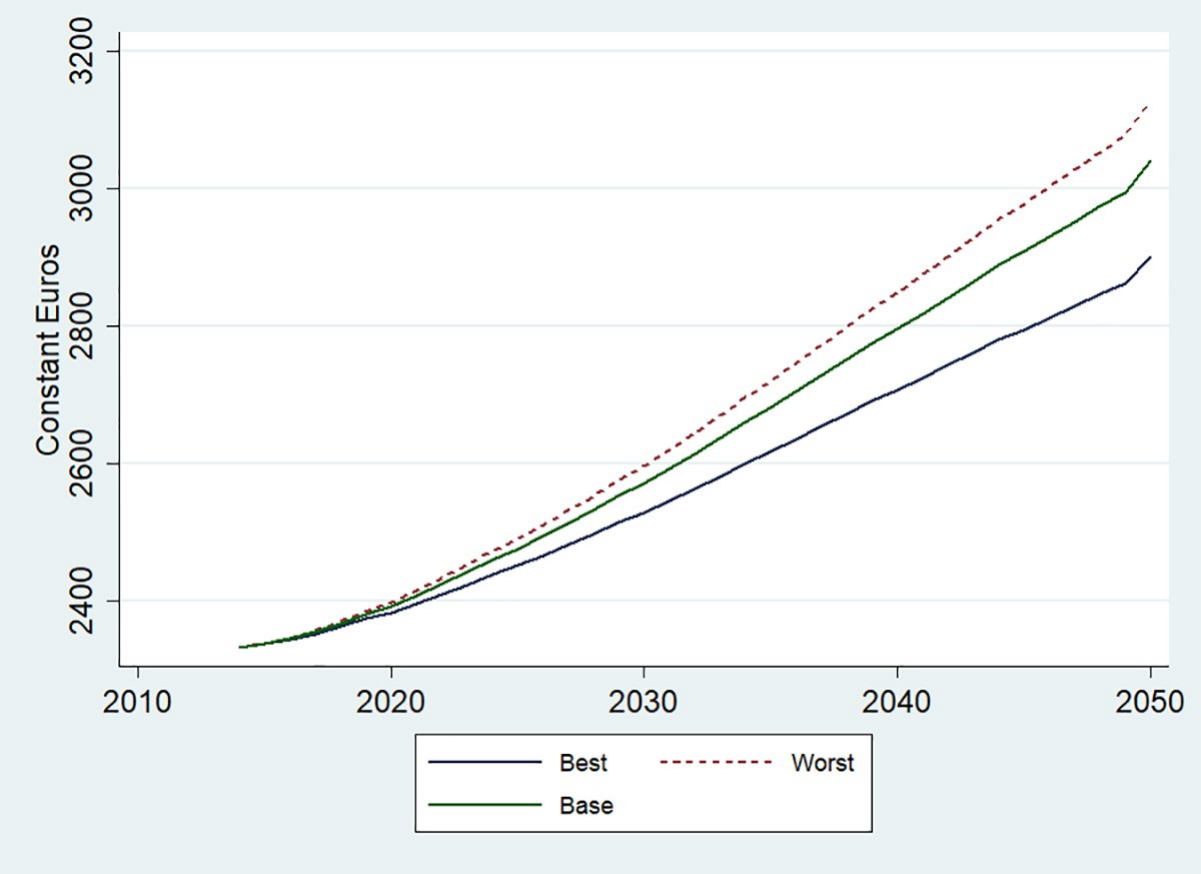
Change in average direct costs in the EU-27 countries and the UK for people 18 years and older, 2014–2050. Base refers to BAU scenario; Best- to BCP and Worst- to WCP.

During the studied period (2014–2050), the population demographic will change, with countries experiencing various trends in their population composition (e.g. in age, gender and size). It is therefore important to look at the impact of the scenarios on predicted medical expenditures per capita. As only the injury costs were available for people younger than 18, we estimated per capita costs for people 18 years of age and older. In all the modelled countries, in the BAU case, these costs are expected to increase by about 30%, from 2,331 Euros in 2014 to 3,040 in 2050 (Fig 2). This increase will be mostly driven by an increasingly ageing population (S5 Appendix in S1 File). At the same time, by 2050, changes in risk factors consistent with the scenarios will lead to a significant deviation from the BAU trend: in the BCP, average cost will be about 4·6% lower than under BAU, and in the WCP- about 2·9% higher. The different trajectories in average predicted costs by region are shown in S4 Appendix in S1 File.

**Fig 3:**
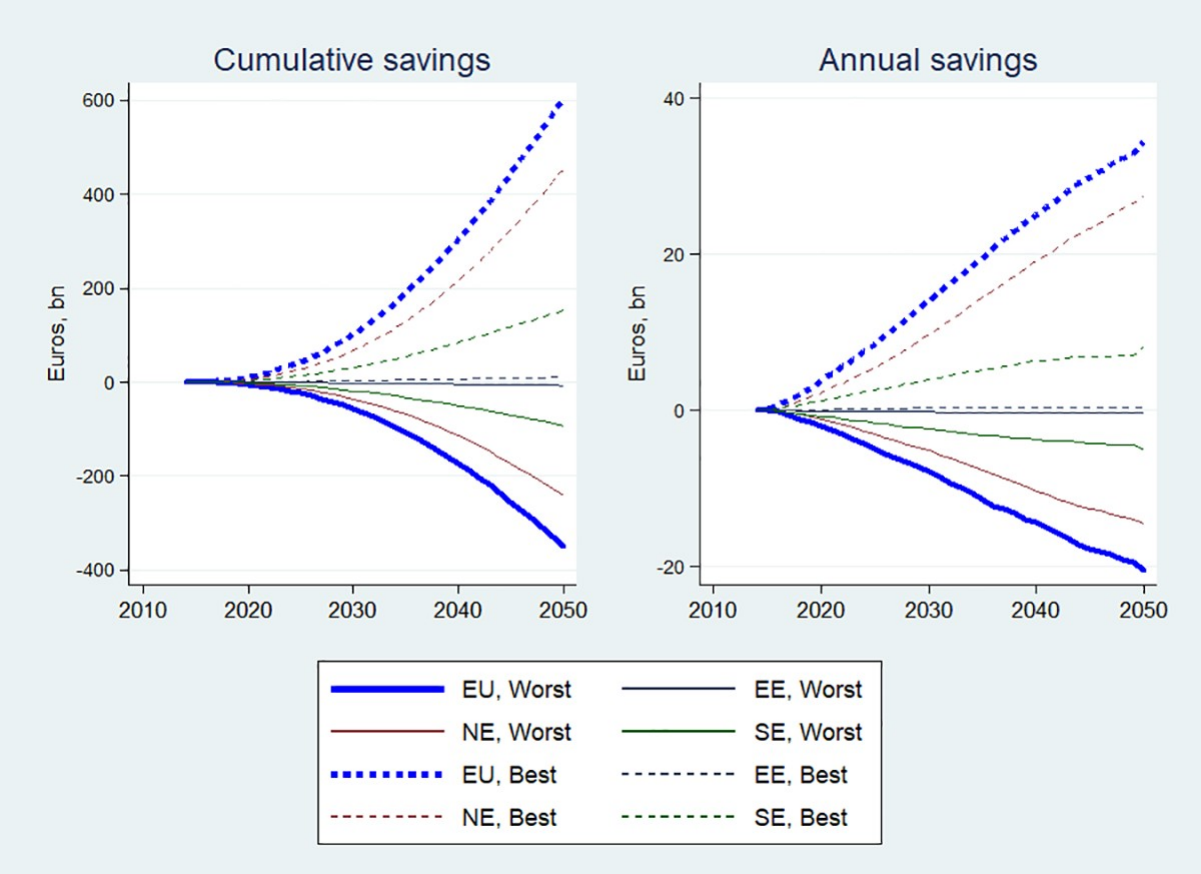
Health expenditure savings compared to baseline scenario, 2014–2050. EE means Central and Eastern Europe; EU: European Union; NE is Northern Europe and SE is Southern Europe.

Cumulatively, 605 billion Euros can be saved by 2050 (or 16.8 billion annually) if the BCP is realized across all the modelled countries compared to the BAU, while excess spending under the WCP is forecast to be about 350 billion Euros (Fig 3, left panel). By looking at specific regions, the largest cumulative savings by 2050 (when comparing BCP to BAU scenarios) can be obtained in Northern Europe (about 453 billion) and Southern Europe (153 billion). The lowest cumulative savings are expected to be obtained in Central and Eastern Europe (about 10.5 billion). Excess spending under the WCP is predicted to be in Northern Europe (242 bn), followed by Southern (92 bn) and Central and Eastern Europe (7·2 bn). The rate of annual savings will accelerate with time, with as much as 34 billion potentially saved in all the modelled countries in 2050, compared to only about 14 billion in 2030 (Fig 3, right panel). Therefore, the economic effect from the improved risk factor profile in the population will take some time materialize, but the savings can be substantial if the long-term view is taken.

**Fig 4:**
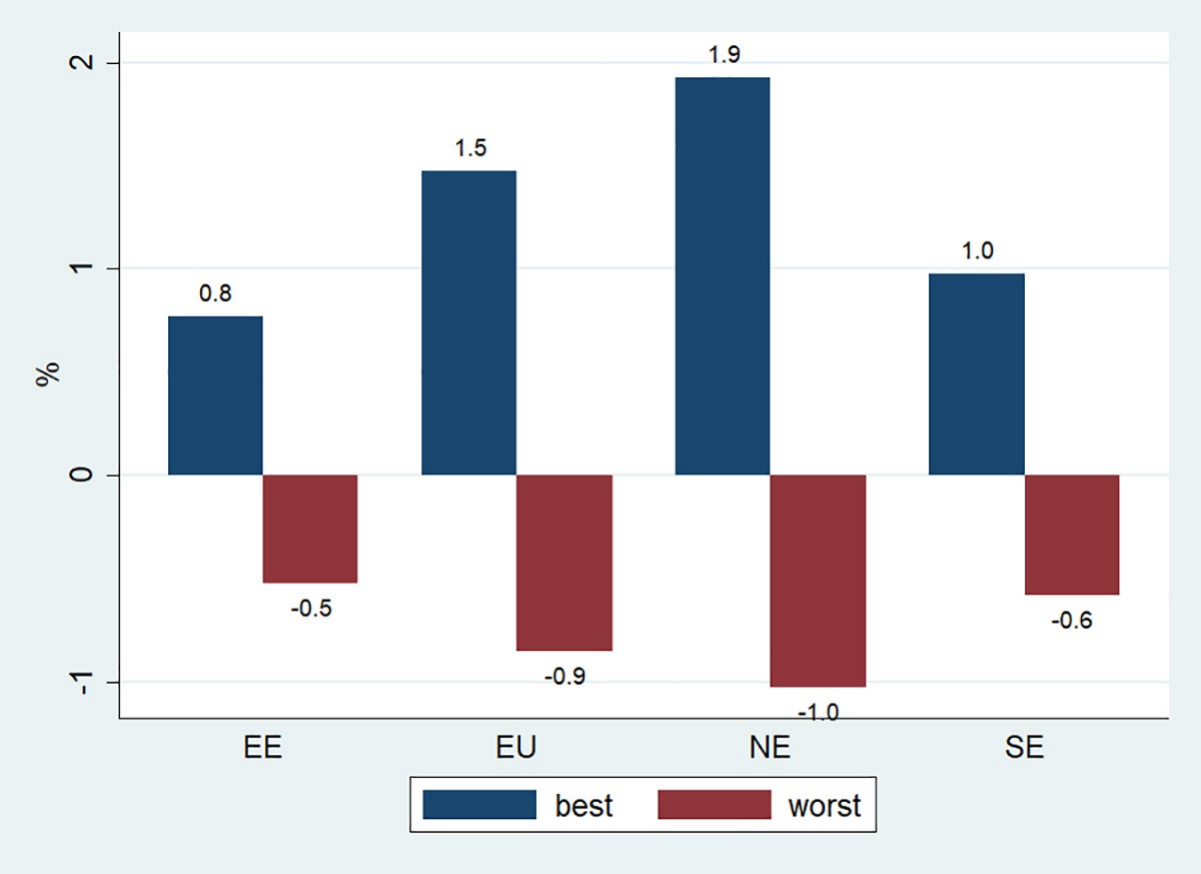
Total savings and losses as a % of total health expenditures, 2014–2050. EE means Central and Eastern Europe; EU: European Union; NE is Northern Europe and SE is Southern Europe. Best refers to BCP and worst- to WCP. Blue bars reprsent savings, red bars-losses.

On Fig 3, the scenario effect on health spending is masked by the differences in the total health spending between regions, which is also a function of the differences in the size of the population and the economies between regions. On Fig 4, one can see that there is much less variability between regions when scenario-specific cumulative health savings/losses are expressed as a proportion of total cumulative regional health expenditures. Nevertheless, it does appear that the savings are somewhat larger in Northern Europe, followed by Southern and then Eastern Europe.

Table 3 suggests that one important reason for this is that the 10 modelled NCDs account for the largest proportion of total health costs in Northern Europe, so that any scenario-specific effect is also likely to be translated into larger savings in that region. In addition, it seems that for one risk factor in particular-hazardous drinking- there is going to be much less improvement under the BCP in Eastern Europe, compared to the other regions (S3 Appendix in S1 File).

**Table 3:** Contribution of the 10 NCDs as % of total healthcare spending, by region and scenario (over 2014–2050).

Region	WCP	BAU	BCP
EU	46.67%	46.00%	44.83%
North	52.81%	52.22%	51.09%
South	36.83%	36.15%	35.01%
East	36.44%	35.83%	34.84%

**Fig 5:**
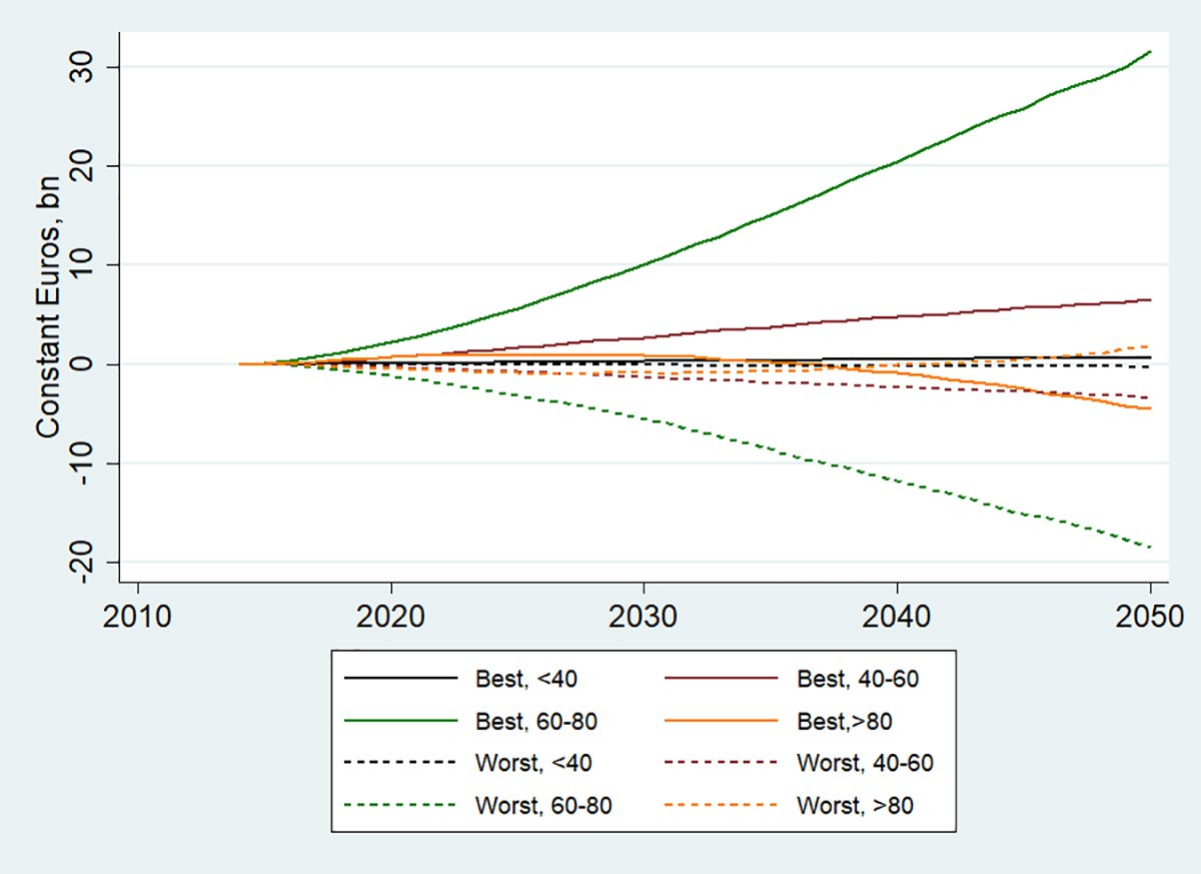
Annual cost savings by age cohorts in the EU, 2014–2050. Best refers to BCP and Worst to WCP.

**Fig 6:**
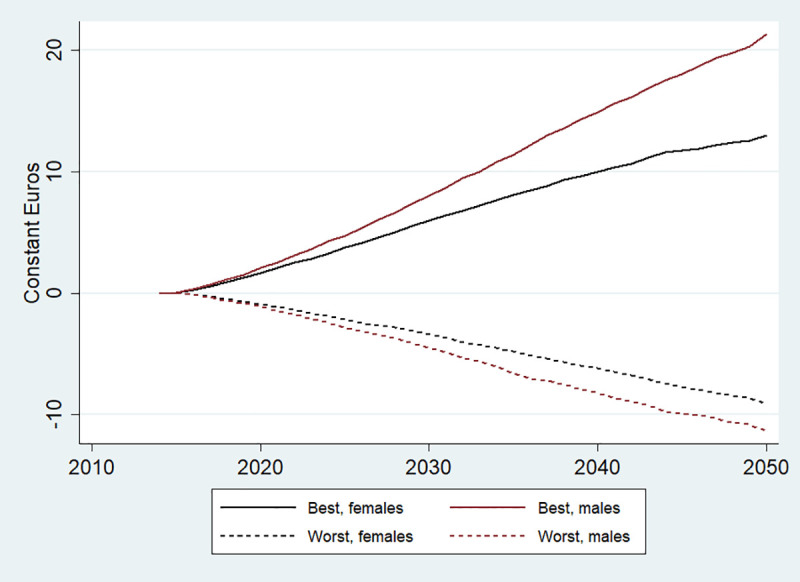
Cost savings by gender in the EU, 2014–2050. Best refers to BCP and Worst to WCP.

Figs 5 and 6 further illustrate scenario-specific cost trends. Thus, older age is generally associated with larger savings and losses (Fig 5). This is not surprising, as diseases associated with the modelled risk factors mostly manifest themselves in the older ages. The only exception is for the oldest age groups, i.e. older than 80, as with time the BCP is expected to lead to cost losses, and the WCP- to cost savings. This can be explained by the fact in this age group, cost savings resulting from better risk factor profiles are outweighed by losses resulting from longer life expectancy.

Finally, Fig 6 suggests that both cost savings and losses depending on the scenarios are expected to be larger for men than for women. This is consistent with the hypothesis that baseline risk factor profile (and health in general) is generally worse among men, so the modelled scenarios are likely to lead to larger epidemiological and cost changes in this group.

## Discussion

As the data availability and modelling capacity improve, there is a growing interest in forecasting future trends in health outcomes and projecting health expenditures. For example, Devaux et al [16] used qualitative and quantitative foresight techniques to project how future societal trends will affect the future burden of NCDs in Europe between 2015 to 2050. Relying on the same microsimulation modelling approach as used in this paper, they concluded that population ageing will be a dominant driver of population health, although the future risk factor trends will also play an important role.

Although a number of studies projecting future health expenditures exist, they tend to be restricted to a single country [28–30]. In addition, they often assume that future risk factor prevalence will remain fixed [29], and rarely take competing risks in individual health histories, as well as patient-level cost data into account, mostly due to the challenges with obtaining the data needed to populate microsimulation models [31,32]. As such, our study has numerous novel features: first, it adopts a life-long perspective on the evolution of the population health, allowing for the fact that people who do not get diseases as a result of an improved risk factor profile may nevertheless accumulate healthcare costs later in life for unrelated reasons. Second, it relaxes the assumption that the risk factor exposure will stay fixed in the future, making it conditional on three plausible scenarios. Third, unlike in most other cost of illness studies, direct disease costs are estimated from medical reimbursement data in a consistent methodological framework, rather than borrowed from a range of disparate sources. Finally, extra cost of comorbidities are explicitly taken into account.

Our micro-epidemiologic simulation exercise shows that the modelled scenarios can nontrivially affect direct medical cost burden in the EU. Depending on a series of alternative assumptions on the distribution and evolution of risk factors across the population, we estimate that by 2050, about 600 billion Euros can be saved cumulatively in the EU-27 and the UK under the BCP compared to BAU, with the amount of savings accelerating over time. In Northern Europe, these savings will be relatively more pronounced: by 2050, total healthcare budgets will be lower there by 3.8% of under BCP compared to BAU, revealing a marked sensitivity of Northern Europe to its current distribution of risk factors.

Nevertheless, total health expenditures (both per capita and aggregate) in the EU-27 and the UK will continue to increase even under the optimistic BCP. The main driver of this will be population ageing, although in Eastern Europe, the effect will be somewhat countered by the emigration- and low birth-rate fuelled population decline.

One of the main strengths of our microsimulation tool is that it allows modelling real-life counterfactuals. For example, in the absence of exposure to smoking, the same individual might still be afflicted with heart disease due to other risk factors, or live longer and continue accumulating healthcare costs for diseases that we are not modelling explicitly. To some extent, this mechanism explains why the BCP does not yield particularly large reduction in spending: people whose morbidity and mortality is reduced however continue to get older and to consume healthcare [24,33]. This lowers the impact of risk-factor specific policy scenarios compared to more traditional cost-of-illness approaches, while at the same time representing a much more realistic cost savings estimates.

The model further contributes to the field of modelling the cost burden of chronic diseases by incorporating the cost of co-morbidities, answering the pertinent question whether there is additionality when two or more disease co-exist in one person^21^. Only a limited amount of high quality results exist in this area, mainly from the US and Nordic countries, and for only a sub-set of conditions.

### Limitations

This analysis only considers the impact of scenarios on direct medical costs. However, it is well known that improvements in health are likely to lead to improvements in other economic outcomes [34]. For example, alcohol-related economic burden may also include direct non-medical costs, e.g. relating to property damage, or “indirect” costs arising from the lost or underutilized labour market resources. Simply put, indirect costs arise when no money physically changes hands, but some resources are still lost/underutilized. We do not estimate such costs in this paper, but they can contribute substantially to the economic impact of the modelled scenarios, often surpassing the direct burden [35]. In addition, although direct costs do contribute to the GDP, in their absence, the money could be invested in other areas, with potentially higher returns, and we do not take this potential “opportunity cost” aspect into account. In the same vein, we also do not take into account the potential cost of policies needed to obtain the variations in risk factors for the two alternate scenarios, which are likely to be very country-dependent. We also do not take into account the potential impact of other variables on the evolution of costs in the future, e.g. the impact of medical technology, which may well differ depending on the scenarios, or possible changes in prices of drugs (e.g. when patents expire). Another limitation is that most available data on the relationships between the modelled risk factors and diseases are from observational studies and therefore causality cannot usually be taken for granted, although the most recent literature we used generally tries to establish reliable causal impacts rather than simple associations. Finally, there were some limitations related to the disease identification in some countries or assumptions behind the cost extrapolation approach.

### Policy implications

All in all, our modelling exercise is generating three important messages. Firstly, unless there is an exceptional effort to reduce risk factor prevalence in Europe which goes beyond the BCP assumption taken in this paper, future health expenditures in this region will be dominated by the population ageing trend, and therefore will continue to grow rapidly. Nevertheless, various population-level preventative interventions may still be cost-effective of even cost-saving, and better value for money than treating a chronic disease once it has developed [36]. Indeed, with the current health system focus in many countries on curative interventions, there is significant room for shifting the focus towards prevention [37], and various innovative intervention packages may be developed to reduce the prevalence of NCDs yet further [38]. In addition, there should be a search for more sustainable modes of care in the future, including greater attention paid to improving rehabilitative and supportive care to keep the elderly independent and well-functioning for as long as possible. Secondly, by taking into account the cost of unrelated competing NCDs as well as co-morbidities, and showing that additionality and super-additionality is significant for a subset of the modelled conditions, the model provides results that not only improve on existing models available to date, but also constitute immediate policy relevance. Super-additionality in disease costs highlights the importance of targeting not only single diseases, but also overall comorbidities, with appropriate policy interventions. The model can provide further guidance about which co-morbidity reduction might result in the highest cost savings. Thirdly, as shown in the accompanying paper [16], although the expected positive effect of an improved risk factor profile is relatively modest for the financial sustainability of health systems, the impact on epidemiological burden as measured by changes in disease incidence, life expectancy and premature mortality is potentially very significant. Likewise, evidence suggests that economic returns to higher labour market productivity may significantly exceed those from healthcare system savings [38]. As the methodological approach we use in this paper does not value this impact in monetary terms (which may include both nonmarket value of improved health, as well as potential improvements in productivity and/or labour force participation), the full economic and welfare benefit from the more favourable risk factor profile is likely to be much greater than the estimated health expenditure saving may suggest.

## Supporting information

S1 File (DOCX)
