# Supplementary material for: Assessing the future medical cost burden for the European health systems under alternative exposure-to-risks scenarios
Source: PLoS One. 2020 Sep 11;15(9):e0238565. doi: 10.1371/journal.pone.0238565 (PMC7485835; doi:10.1371/journal.pone.0238565)
Supplement: S1 File — (DOCX) [file pone.0238565.s001.docx]

# S1 Appendix. Modelling direct costs – general estimation approach

## Introduction

The annual per capita direct medical costs were estimated using the administrative health expenditure datasets from three countries-France, Estonia and the Netherlands. These costs were then extrapolated to all remaining EU-27 countries and the UK (for more details about the extrapolation methodology, see section d below). In this section, we describe the overall approach to estimating the extra costs of diseases and comorbidities in all three countries. For country-specific issues, see the country reports located at [1–3].

The goal of the estimation approach described in this section is to predict the total healthcare costs, for each patient, conditional on age, gender, and the disease status. The general cost formula is as follows:

$Cost_{i,total}=Cost_{i,residual}+Cost{}_{i,extra-main}+Cost_{i,extra-comorb}+Cost_{i,extra-death}$ (1)

For a person *i* the total costs *Cost _i, total_* will comprise the costs that either are explicitly modelled (e.g., for treating cancers or diabetes), or those that are not. This latter component, which is called residual cost *Cost _i, residual_* in our model, will capture costs assumed to be unrelated to the high BMI, low physical activity or harmful alcohol consumption risk factors, capturing, for example, the costs of treating migraine or common colds, among many others. If people have only one microsimulation-defined disease, their total cost will be equal to the sum of their predicted residual cost and the predicted extra cost of having this disease. If people have two diseases, the comorbidity cost component (see Box S1) will also be added to this sum, as explained below. The people may also die over the observation period, in which case their total predicted costs should also include death-related healthcare expenditures (over and above other costs). Due to the data limitations, the comorbidity component does not take into account more than two explicit diseases. In cases where a person in the model develops more than two diseases, the two most recent ones are kept, and the older ones are discarded.

| **Box S1. Defining main diseases and comorbidities**  For our modelling purposes, we assume that the “main disease” is the most recently diagnosed one, and the “comorbidity” is a disease that was diagnosed earlier. For example, if a person has had diabetes for several years, and was diagnosed with cancer this year, then we would first estimate the extra cost of having a “main disease”- cancer (in the sample with comorbidities), and then estimate the extra cost of having a comorbidity- i.e. diabetes in the presence of cancer. |
| --- |

In principle, one could predict the total healthcare costs *Cost _i, total_* (shown in formula 1 above)– also including out-of-pocket spending – for each person *i* by estimating the parameters in the two-way interaction model (for both genders separately) as described in [1]:

$\ln\left( {Cost}_{i} \right)=\alpha+\beta\cdot{age}_{i}+\gamma_{k}\cdot D_{i,k}+\gamma_{j}\cdot D_{i,j}+\gamma_{kj}\cdot D_{i,k}\cdot D_{i,j}+\varepsilon_{i}$ (2)

Where Cost_i_ is a total medical cost, defined as follows:

*“In a bottom-up design, units of health care are used on a patient level and are multiplied with a price for this unit. All individual health expenditures are then summed up to calculate total cost of the disease* [4].

In addition, age_i_ corresponds to age; $D_{i,k}=1$ if individual $i$ suffers from illness $k$, =0 otherwise, and ε_i_ is an error term assumed to be uncorrelated with the covariates of interest. The intercept α represents the predicted medical cost for a person aged 18-39, without any diagnosed modelled disease (=residual cost).

Model (2) can be estimated using the sample of people with positive costs using the generalized linear model estimator, based on the multivariate gamma regression with a log link (see [5] for the choice on appropriate econometric specification for France). This is a frequently used approach to model highly skewed data such as medical expenditures, whereby the so-called index function based on the covariates of interest is exponentiated via the log link to allow the non-negative prediction of the medical costs. Such an approach has an advantage over, for example, the ordinary least squares (OLS) estimator, as it avoids the need for retransformation when the goal is to predict actual, rather than log-transformed expenditures, as well as taking heteroscedasticity into account [6].

For example, the predicted average cost for people aged 55 with no modelled diseases and with positive costs would be equal to:

E(C|C>0)= $exp(\hat{\alpha}+\hat{\beta}_{50-55})$

For a person with diagnosed[[1]](#footnote-1) diabetes of the same age, the total predicted cost in this sample would be equal to:

E(C|C>0, diabetes=1)= $exp(\hat{\alpha}+\hat{\beta}_{50-55}+\hat{\gamma}_{diabetes}$)

For a person with both diabetes and cancer, the total cost in the sample of people with positive costs can be predicted as:

E(C|C>0, diabetes=1; cancer=1)= $exp(\hat{\alpha}+\hat{\beta}_{50-55}+\hat{\gamma}_{diabetes}+\hat{\gamma}_{cancer}+\hat{\gamma}_{cancer*diabetes}$)

To make sure that these predicted costs are representative not just of the people with positive medical costs, but of all the people with the diagnosed conditions, an adjustment should be made by multiplying these costs by the probability of having positive medical costs. This adjustment is necessary because not all diagnosed people will incur positive medical expenditures. For example, the total predicted cost for a person with diabetes is:

E(C|diabetes=1)= P(C>0)*E(C|C>0, diabetes=1)

The first part[[2]](#footnote-2) of this two-part model estimator can be estimated using logit regression:

$$P(C>0)=\Phi(a+b \cdot AGEcat)$$

where $\Phi$ is the cumulative standard logistic distribution function.

The practical problem with estimating model (2) is that the sample size for several conditions in the analysed datasets is too small for two-part model estimation with interactions. Predicting costs using model (2) is even more problematic if one wants to take into account the information on the length of time since diagnosis contained, for example, in the French dataset. Therefore, it was decided that the total medical costs in France (and, for consistency, in all other countries) will be predicted by separately estimating each component listed in equation (1), as described in the sections that follow.

## Cost component estimation

As discussed in [1], extra disease costs are estimated as the mean marginal difference of the predicted outcome with a disease variable switched on or off. There are two different (but related) approaches, depending on whether there was chronic disease comorbidity.

In general, average medical cost for any age and gender can be predicted as follows:

𝐸(C) = 𝑃(C > 0)*E(C|C > 0)+ 𝑃(C=0)*𝐸(C|C = 0) = 𝑃(C > 0)*𝐸(C|C > 0) (3)

The average extra cost of a disease for diagnosed people can therefore be estimated, for a given gender and age group, as the difference in the predicted costs, conditional on the disease status:

$\hat{Cost}_{extra}$= 𝑃(C>0|disease=1)*E(C|C >0,disease=1)- 𝑃(C>0|disease=0)*𝐸(C|C>0,disease=0) (4)

Another way to think about the first part of this formula is that E(C|C>0,disease=1) component is representative of the population with a diagnosed disease who have positive medical expenditures, while multiplication by 𝑃(C>0|disease=1) factor makes such costs representative of the medically diagnosed population with a disease (who may or may not have positive medical expenditures).

Estimating the first part (i.e. the probability) components of the two-part model (4) is however complicated, because for a number of diseases in France (and for all diseases in Estonia and in the Netherlands), the disease definition in the administrative data depended on whether positive costs were reported. Therefore, estimating 𝑃(C>0|disease=1) with a logit or a similar approach was generally impossible. Even when this was not strictly the case (i.e. when a small proportion of patients with a disease had zero costs), estimating P(C > 0|disease=1) was not a feasible option because the disease definition was strongly endogenous to the probability of having nonzero expenditures. Therefore, it was decided to estimate the extra costs of diseases using the following formula:

(5)

As one can see, the first part probability is predicted unconditional of the disease status (but conditional of age). This is not ideal, because the probability of having non-zero costs is likely to be higher in the sample of sick people than in the sample of healthy people. To deal with this, one could have assumed, for example, that the probability of having non-zero expenditures was equal to 1 in the sample of people with a disease. However, this assumption is arbitrary and it might actually lead to cost overestimation. On the other hand, our decision to use 𝑃(C>0) probability in the first part is likely to lead to a conservative extra cost estimation.

In any case, our estimates suggest that the difference between these probabilities is relatively small in the middle ages and for the elderly (i.e., in the 50-90 y.o group), especially among women. In the samples with at least one comorbidity, there is very little difference in the predicted probabilities depending on the main disease status.

Equation (5) was estimated in two samples, by age and gender:

a) Without any other chronic disease (i.e., predicted average costs were compared among patients with a disease and without a disease, in the sample with no other chronic diseases), and

b) With at least one other chronic disease, the predicted costs for patients without a disease of interest were subtracted from predicted costs for patients with a disease of interest.

The parameters in the second part of the two-part model as described by equation (5) were estimated similar to model (2), but without the interactions, and with a dummy for a given disease of interest (rather than for a vector of diseases):

$\ln\left( {Cost}_{i} \right)=\alpha+\beta\cdot{age}_{i}+\gamma_{k}\cdot D_{i,k}+\varepsilon_{i}$ (6)

For example, the extra cost of diabetes for a woman aged 55 is predicted using parameters estimated in model (5) as follows (separately for samples with and without any comorbidities):

$$=\Phi(\hat{a}+\hat{b}_{50-55})\times\left[ \exp\left( \hat{\alpha}+\hat{\beta}_{50-55}+\gamma_{diabetes} \right)-\exp(\hat{\alpha}+\hat{\beta}_{50-55}) \right]$$

$$\Longleftrightarrow$$

$\hat{Cost}_{extra}=\frac{\exp(\hat{a}+\hat{b}_{50-55})}{1+(\hat{a}+\hat{b}_{50-55})}\times\left[ \exp\left( \hat{\alpha}+\hat{\beta}_{50-55}+\gamma_{diabetes} \right)-\exp(\hat{\alpha}+\hat{\beta}_{50-55}) \right]$ (7)

As one can see from equation (7), the effect of having a disease on predicted extra medical costs is nonlinear and depends on the age category.

The extra (extra) cost of comorbidity is estimated as the difference in the predicted costs for patients with both the “main” disease and comorbidity (as defined above) versus the predicted cost for patients with just a “main” disease:

(8)

The parameters for this equation are estimated using a model similar to (2) shown above, i.e. including not only the main disease dummies, but also their interactions. However, as already mentioned, we could not estimate this model for every single disease, nor could we take into account the length of time since diagnosis because of the sample size limitations. Therefore at this step, we had to combine several diseases into groups (e.g. this applied to both types of strokes and to cancers). In addition, to reduce potential for residual confounding, a dummy was also included to control for long-term chronic diseases (not specifically modelled) in the case of Estonia and France analyses.

Finally, the estimation approach for the extra cost of death differed in the three countries. In the French case, extra cost of death was estimated using a similar two-part modelling approach, as the mean marginal difference of the predicted outcome (total health expenditure in 2014) with a death dummy (corresponding to dying in the first semester of 2015) switched on or off. As the data on costs accumulated throughout 2014 was available, and the information on whether a person died was provided for 2015 as well, this difference was estimated for people who died in the first semester of 2015, compared to the people who stayed alive in the same period. Including people who died in the second semester of 2015 in the analysis was ruled out because the date of death for them was too far away from 2014, and therefore it was likely that the extra cost of death would be underestimated for them.

We conducted this analysis separately for two samples: with at least one model-defined NCD, and without any NCDs. These costs were then added on top of the other healthcare expenditures. To avoid the issue of potential double-counting of the death-related costs, the main cost analysis was restricted to the people who were still alive on 31 December 2014, and therefore proportion of those who died in the first semester of 2015 was small in relation to the total. In Estonia, and the Netherlands, it was impossible to estimate extra deaths costs using this approach due to data limitations, and therefore for all countries the extra costs of death were extrapolated from the French ones using the approach described below.

In the microsimulation model, we assumed that the people who died accumulated only half of their extra residual and disease costs in the last year of their lives life.

## Residual cost and related issues

We estimate age- and gender-specific average residual costs by restricting the sample to people who had no model- defined diagnosed diseases (but could have other diagnosed conditions, including chronic ones, or were otherwise healthy). Such people may or may not have had zero health expenditures.

A potential complication is that in our microsimulation model, people are assigned disease status based mostly on IHME epidemiological prevalence (with some additional calibrations as appropriate), which may well be different from the administrative dataset-based prevalence derived from medical diagnoses and/or drug use. In such datasets, people with undiagnosed health conditions may be misclassified as not having the conditions that we are modelling, and therefore their costs will be inappropriately part of the residual costs. Such costs will also not be captured when estimating the extra cost of disease.

One potential way to deal with this is to assume that IHME-based prevalence reflects “correct” epidemiological prevalence (i.e. including both diagnosed and undiagnosed cases). Under this assumption, we could in theory adjust the predicted costs of disease by multiplying it by some factor based on the difference between “diagnosed” and “real” prevalence. If we find, for example, that the prevalence of diabetes based on administrative data is 10%, while IHME-based prevalence is 12%, then we could multiply the estimated extra cost of disease in this group by 10/12=0.83, to make sure that such costs are representative of women who are both diagnosed and undiagnosed. Alternatively, we could assume that the extra disease cost equals zero for the proportion of people who is undiagnosed according to IHME data. Likewise, we could re-categorize our residual costs accordingly, which is likely to increase residual costs because a number of “residual” observations with low/zero expenditures will be reduced. Therefore the net effect on the total costs is ambiguous.

Nevertheless, it is not certain that IHME-estimated disease prevalence is necessarily superior to the administratively-derived one, as it relies on data of varying quality and methodological basis (e.g. it can be based on multiple sources of survey data, with additional assumptions to correct for self-reporting bias). Some analysis (see below) shows that for example in France, age and gender-specific prevalence of diabetes and of several cancers is higher in the administrative than in the IHME dataset, which suggests this divergence may not be due to the inclusion of undiagnosed cases in IHME data. Although in some other cases, the prevalence was considerably higher in the IHME dataset, this was mostly true at the oldest and the youngest ages, where IHME estimation methodology might rely on too little data and on too many assumptions. In addition, at the oldest ages (generally older than 60-70), where the prevalence rates diverge the most, the absolute numbers of affected people gets lower with each year of life, therefore the total impact on costs is more limited than the actual graph may suggest. Therefore, we prefer not to further adjust the extra disease/residual costs. Besides, since we are interested mostly in the “delta effect” of different interventions/scenario comparisons, the potential overestimation issue stemming from assigning the estimated costs to the undiagnosed cases is probably of minor significance.

## Cost extrapolation to other countries

Our extrapolation methodology is based on the assumption that the annual treatment cost differentials between countries are time-invariant[[3]](#footnote-3), and that they are mostly driven by the differences in two components: cost per unit of treatment received, as well as the population-level intensity of treatment provided. For example, the spending per capita for inpatient costs can be broken down as follows:

(9)

Where $\frac{Spending}{Disch\arg e}$can be viewed as the average price per unit of inpatient treatment received, while$\frac{Disch\arg es}{Capita}$ represents the population-level “intensity” of hospitalization received in a given country. Then, one can divide spending per capita for inpatient treatment in country *a* by spending per capita in country *b*, and multiply the costs in an anchor country by this ratio to extrapolate to a comparator country.

Given that the cost of treatment is also determined by other components, we also estimated $\frac{Spending}{Capita}$ ratios between countries for outpatient and pharmaceutical costs. For example, for outpatient costs, this ratio can be viewed with similar interpretation:

(10)

To estimate these differentials, we used the OECD data[[4]](#footnote-4) on the inpatient curative and rehabilitative care spending/capita; outpatient curative and rehabilitative care spending/capita; medical goods spending/capita. To ensure comparison and to smooth over temporary data variability, we used this data averaged over 3 years: 2012, 2013 and 2014, expressed in current prices.

After estimating the differentials in these three components, we take them all into account when estimating the overall disease-specific conversion factor between countries. It’s possible, for example, that diseases in the more acute stage, such as myocardial infarctions and strokes, have a much greater inpatient component than for example diabetes. Therefore, the overall disease-specific conversion factor to extrapolate between countries can be represented by the formula:

Conversion factor = (11)

Where $x_{i}$ is the ratio of per capita inpatient; outpatient or pharmaceutical spending between countries (based on OECD data); $w_{i}$are the weights assumed to represent treatment proportions for each disease. These weights were also obtained based on the OECD SHA data on the expenditure by disease, in millions national currency units. For example, to extrapolate the costs from the Netherlands to other countries in Northern/Southern Europe, the following was estimated (Table S1):

**Table S1. Treatment component weights by disease, Netherlands (Southern and Northern region extrapolation)**

| Disease | Inpatient, % | Outpatient, % | Pharma, % |
| --- | --- | --- | --- |
| Circulatory system  (Both strokes, MI) | 62.2% | 18.4% | 19.4% |
| Neoplasms  (Cancers) | 71.0% | 15.9% | 13.1% |
| Endocrine and metabolic diseases  (Diabetes) | 16.0% | 30.1% | 53.9% |
| Genitourinary diseases  (CKD) | 58.7% | 16.4% | 24.9% |
| Respiratory diseases  (COPD) | 38.4% | 19.2% | 42.4% |
| Digestive system diseases  (Cirrhosis) | 25.4% | 58.6% | 16.0% |
| Mental disorders  (Depression) | 83.2% | 10.9% | 5.9% |
| Nervous system diseases  (Neurologic disorder) | 48.5% | 13.4% | 38.0% |
| Injury/poisoning  (Alcohol disorder) | 67.0% | 29.2% | 3.8% |
| Remaining costs | 33.3% | 33.3% | 33.3% |
| Death costs | 33.3% | 33.3% | 33.3% |

*Source*: <http://stats.oecd.org>

Note: In the first column, diagnostic category as defined in SHA framework is indicated, followed by the (disease(s)), to which this category applies. For example, the distribution of costs for CKD (belonging to the genitourinary category) is assumed to be split as follows: 58.7% for inpatient care; 16.4%- outpatient care and 24.9%- pharmaceutical care.

Of interest are the proportions in the last 3 columns. As one can see, for conditions such as strokes, MI, alcohol disorders, cancer and depression, inpatient costs indeed account for a large proportion of total medical costs. On the other hand, for diseases that are more chronic in nature, such as diabetes and COPD, pharmaceutical component plays a greater role. For simplicity (and due to the lack of information), we assumed that the differentials in residual and death-related costs between countries were equally driven by the differentials in the three separate treatment components. In addition, we assumed that in the first year of diagnosis, the differentials in more acute conditions such as strokes, MI and cancer, are driven entirely by the differentials in hospitalization costs. In Table S2 below, three examples for the extrapolation factors are shown where the costs are extrapolated from the anchoring countries (France, Estonia and the Netherlands) to three other countries (Italy, Bulgaria and Austria). For example, in the first year after diagnosis, the MI costs in France will be multiplied by 0.68 to predict the costs for this condition in the first year after diagnosis in Italy. In the second year, the adjusting factor for this disease and this pair of countries is equal to 0.70. As mentioned in the preceding paragraph, the first year extrapolating factors for the acute conditions such as MI are different for the second (and follow-up) year ones, as we make the assumption that acute condition cost differentials are entirely driven by the hospitalisation cost differences in the first year.

**Table S2 Extrapolating factors from anchoring countries: 3 examples**

| Disease | 1st year | 2nd year+ |
| --- | --- | --- |
| Southern EU: France to Italy | | |
| Ischemic stroke | 0.68 | 0.70 |
| Hemorrhagic stroke | 0.68 | 0.70 |
| MI | 0.68 | 0.70 |
| Cancers | 0.68 | 0.70 |
| Diabetes | 0.70 | 0.70 |
| CKD | 0.69 | 0.69 |
| COPD | 0.69 | 0.69 |
| Cirrhosis | 0.74 | 0.74 |
| Depression | 0.69 | 0.69 |
| Neurologic disorder | 0.69 | 0.69 |
| Alcohol disorder | 0.71 | 0.71 |
| Remaining costs | 0.71 | 0.71 |
| Death costs | 0.71 | - |
| Eastern EU: Estonia to Bulgaria | | |
| Ischemic stroke | 0.89 | 1.12 |
| Hemorrhagic stroke | 0.89 | 1.12 |
| MI | 0.89 | 1.12 |
| Cancers | 0.89 | 1.10 |
| Diabetes | 1.25 | 1.25 |
| CKD | 1.01 | 1.01 |
| COPD | 1.08 | 1.08 |
| Cirrhosis | 1.00 | 1.00 |
| Depression | 1.10 | 1.10 |
| Neurologic disorder | 1.13 | 1.13 |
| Alcohol disorder | 0.89 | 0.89 |
| Remaining costs | 0.98 | 0.98 |
| Death costs | 0.98 | - |
| Western EU: Netherlands to Austria | | |
| Ischemic stroke | 1.63 | 1.41 |
| Hemorrhagic stroke | 1.63 | 1.41 |
| MI | 1.63 | 1.41 |
| Cancers | 1.63 | 1.46 |
| Diabetes | 1.19 | 1.19 |
| CKD | 1.41 | 1.41 |
| COPD | 1.32 | 1.32 |
| Cirrhosis | 1.13 | 1.13 |
| Depression | 1.52 | 1.52 |
| Neurologic disorder | 1.38 | 1.38 |
| Alcohol disorder | 1.39 | 1.39 |
| Remaining costs | 1.25 | 1.25 |
| Death costs | 1.25 | - |

*Note*: For example, in the first year after diagnosis, the MI costs in France will be multiplied by 0.68 to predict the costs for this condition in the first year after diagnosis in Italy. In the second year, the predicting coefficient is equal to 0.70. MI: myocardial infarction; CKD: chronic kidney disease; COPD: chronic obstructive pulmonary disease.

# S2 Appendix. Injury costs

The costs of some of the micro-simulated conditions (e.g. back pain and injuries) were impossible to estimate using the administrative data. For injury costs, a combinatorial approach between a PAF-, microsimulation-, and bottom-up approach was applied. Of interest to us is not the average cost of all injuries regardless of origin, but rather the cost of specific injuries related to the most important risk factors- in particular alcohol consumption (as this risk factor is potentially amenable to various policy interventions that we can simulate in our model). As shown in Table S3, road, unintentional injuries and self-harm comprise some of the conditions that together represent 95% of the alcohol-related disease burden. We also assume that inter-personal violence is likely to be related to harmful alcohol consumption in many cases.

**Table S3. Conditions associated with 95% of the alcohol-related disease burden in the EU27+UK**

| Cirrhosis |
| --- |
| AUD |
| Road and unintentional injuries |
| Self-harm |
| Liver Cancer |
| Breast Cancer |
| CVD |
| Nasopharynx & Oesophageal Cancer |
| *IHD** |
| *DM** |
|  |

1. *Note*: Note that (moderate) alcohol use is protective of IHD (Ischaemic Heart Disease) and DM (Diabetes Mellitus) based on GBD 2016. Conditions are listed in descending order of contributing to the disease burden.
2. So*urce:* OECD analysis of Global Burden of Disease 2016 data

The data on the associated ICD-10/ISHMT codes was available for several countries (see Table S4 for the list of countries which provided injury health spending data per five-year age group, and Table S5 to see the matrix we used to convert the country-provided ICHTM codes into the GDB sequela). Separately, we also estimated the cost of lower back pain- a condition likely to be related to obesity- but this cost will be implemented in the future model iteration.

Specifically, the estimation of the alcohol-related injury costs involves the following three components:

- Estimation of the per alcohol-related case injury costs (per age, country, sex)
- Estimation of alcohol-related injury incidence (per age, country, sex) under various policy scenarios
- Combination of the per-case injury cost with incidence estimates, to estimate the national injury costs attributable to alcohol use

**Table S4. Type of injury costs obtained from countries**

| Country | Data level and coding | Type of care | Year data available |
| --- | --- | --- | --- |
| Estonia | ICD-10 coding, by age and sex | All providers, no drug costs | 2015 |
| Sweden | ISHMT coding, by age and sex | Inpatient Injury treatment costs | 2016 |
| Greece | Average injury costs, by age and sex | All providers | 2013 |
| Hungary | Average injury costs, by age and sex |  | 2014 |

*1. Note*: This table highlights progress made on retrieved data from country statistics offices, reflecting the cost of injury by age and sex, broken down into more detailed injury types and providers, where data are available.

*2. Source*: OECD 2017/2018

The injury cost data provided was either an average cost per case, age group, and sex (i.e. Greece), or a cost per treatment, age, sex, and ICD-Code (Estonia), or based on ISHMT injury groups (e.g. intracranial injury). The ‘per case’ cost refers to the sum of costs incurred by the same individual under the same diagnosis over the span of one year. For example, if someone incurred an ankle injury and sought multiple physiotherapy treatment sessions, the ‘per case’ cost for the ankle injury reflects the sum of these multiple visits. ‘Per treatment’ cost would only reflect the cost for one single session. Both ISHMT-codes as well as ICD-10 codes can be related through a set of matrices and weights to these IHME groups. Table S5 shows the relationship between ISHMT codes and IHME injury sequelae. This information was then applied to the obtained cost of injury data to obtain the per case, age group, and sex specific injury costs that are then applied to the injury cases.

**Table S5 Conversion between ISHTM codes and GBD sequelae for alcohol attributable injuries**

|  | 1901 | 1902 | 1903 | 1904 | 1905 | 1906 | 1907 | 1908 | 1909 | 1910 | 1911 |
| --- | --- | --- | --- | --- | --- | --- | --- | --- | --- | --- | --- |
| GBD Sequaelae | Intercranial injury | Other injuries to the head | Fracture of forearm | Fracture of femur* | Fracture of lower leg | Other injuries | Burns and corrosion | Poisonings | Complications of surgical medical care | Sequelae of injuries | Other unpsecified effects of external causes |
| Road Injury | x | x | x | x | x | x |  |  | x | x | x |
| Unintentional other transport injury | x | x | x | x | x | x |  |  | x | x | x |
| Unintentional falls | x | x | x | x | x | x |  |  | x | x | x |
| Unintentional drowning |  | x |  |  |  | x |  |  | x | x | x |
| Intentional self harm |  | x |  |  |  | x |  | x | x | x | x |
| Interpersonal violence | x | x |  |  |  | x |  | x | x | x | x |
|  |  |  |  |  |  |  |  |  |  |  |  |
| ICD10 | S06 | S00-05, S07-09 | S52 | S72 | S82 | S10-S51, S53-S71, S73-S81, S83-T14, T79 | T20-T32 | T36-T65 | T80-88 | T90-T98 | Remainder of S00-T98 |
|  |  |  |  |  |  |  |  |  |  |  |  |
|  |  |  |  |  |  |  |  |  |  |  |  |
| Cutoff for Sequelae conversion: >3% |  |  |  |  |  |  |  |  |  |  |  |
| GBD Sequaelae | N27 | N1, N2, N3 | N7 | N8 | N10 | N1, N2, | N14, N16 | N2 | N2 | N23, N28, N21, N16, N6 | |

* fracture of femure <3% of all road injury, transport injury, and unintentional falls** Burns and corrosion: all <3% for alcohol related injuries *Source*: OECD 2018

We assume that all people with injuries (as per IHME-generated incidence data) will get treated and therefore incur a positive cost. This is probably a reasonable assumption given the nature of the injuries we are modelling. Even in the cases of drowning/self with lethal outcome, considerable expenditures can occur.

Since not all countries have cost data for injuries available, the extrapolation methods described above are used. The extrapolation component calibrated the costs based on inpatient outpatient and pharmaceutical expenditure ratios, and rescaled the costs to reflect total population coverage. These data was then combined with injury case outputs under various policy scenarios, obtained from the microsimulation model. Also, note that the costs of injuries were estimated for people of all ages, while for other diseases the data was only available for people aged 18 and older.

# S3 Appendix. Change in average risk factor prevalence over time


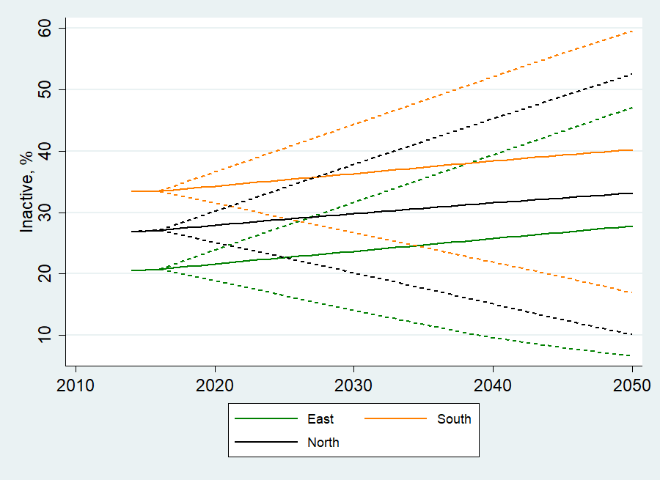


**Fig S31. Change in inactivity prevalence, 2014-2050**


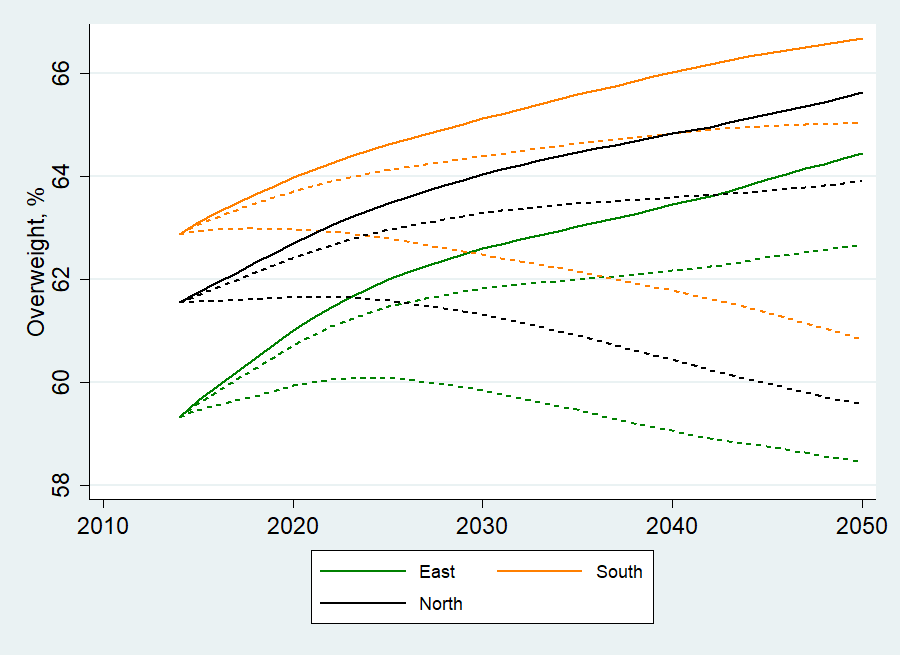


**Fig S32 Change in overweight prevalence, 2014-2050**


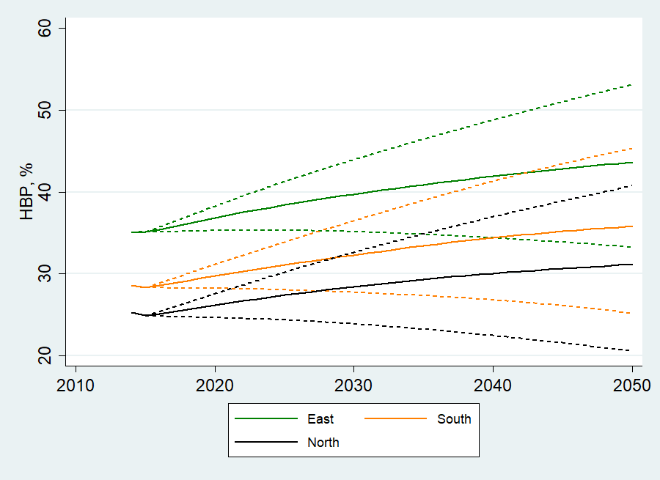


**Fig S33 Change in HBP prevalence, 2014-2050**


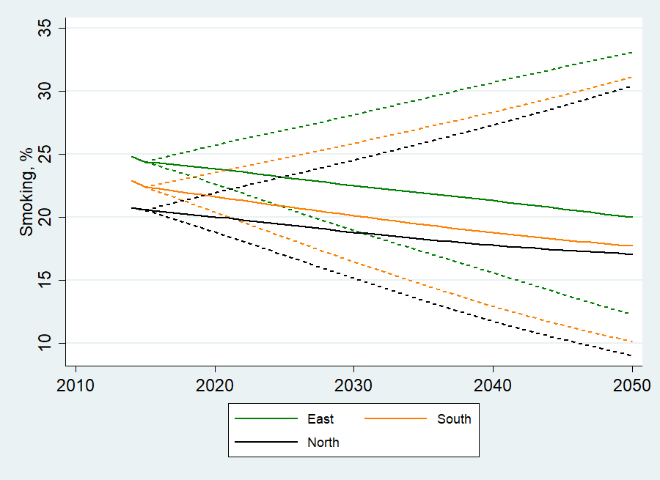


**Fig S34 Change in smoking prevalence, 2014-2050**


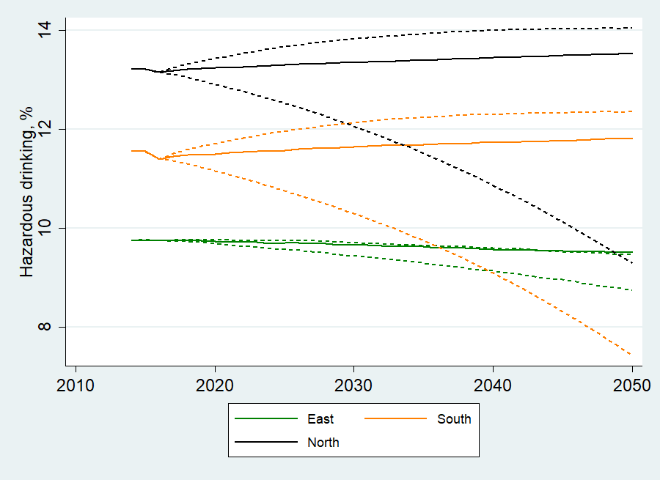


**Fig S35 Change in hazard drinking prevalence, 2014-2050**

# S4 Appendix. Change in average direct costs for people 18 years and older by region, 2014-2050


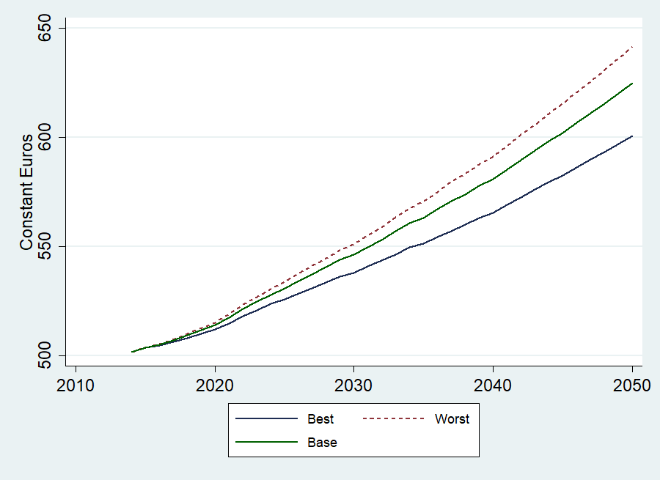


**Fig S41. Change in COI per capita in EE, 2014-2050**


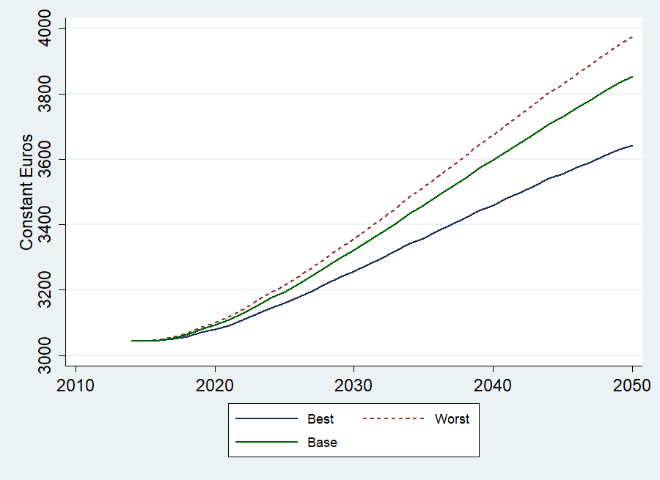


**Fig S42. Change in COI per capita in NE, 2014-2050**


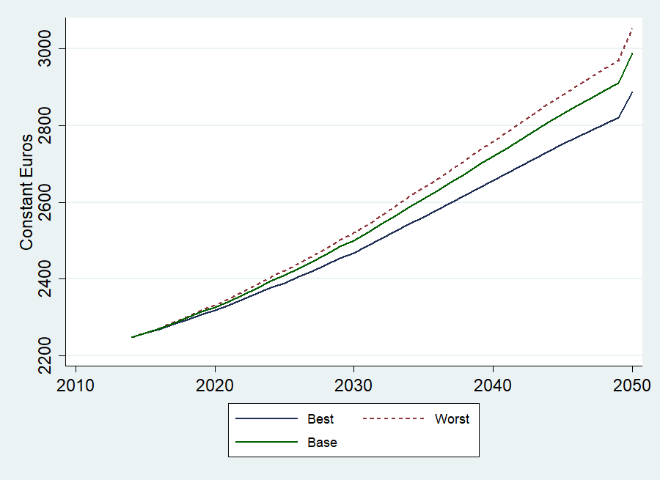


**Fig S43. Change in COI per capita in SE, 2014-2050**

# S5 Appendix. Projected demographic changes


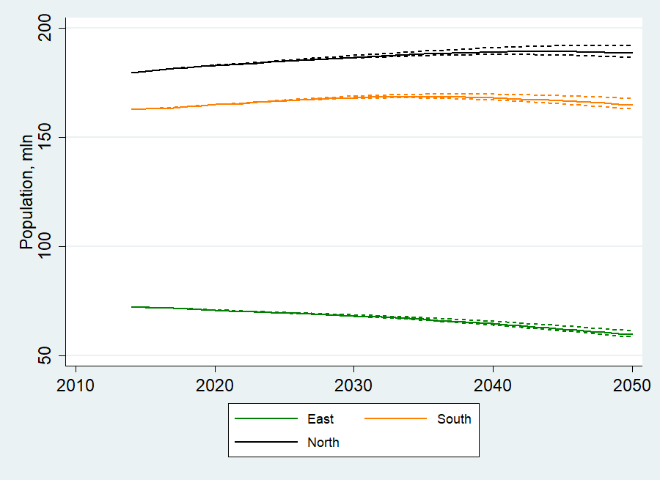


**Fig S51. Change in population by region and scenario, 2014-2050**


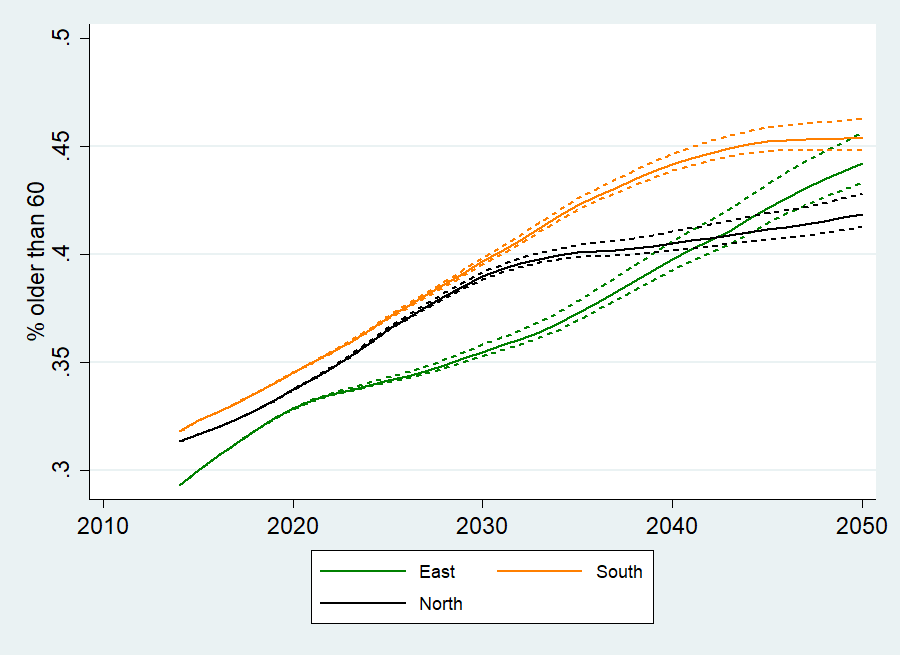


**Fig S52. Change in proportion older than 60 by region and scenario, 2014-2050**

# S6 Appendix. Additional information on the data sources used in the model

This appendix is based on the accompanying paper [7].

The general framework of the model is presented in Cecchini et al [8] and Devaux et al [9]. A full documentation of the model structure is made freely available online, including a report on the demography module [4] and a report on the diseases and risk factors module [8].

The data sources for diseases and risk factors exposure for smoking and physical inactivity use the IHME Global Burden of Diseases (GBD) study, 2016 [10]. Alcohol consumption is modelled using the level of alcohol consumption from the WHO Global Health Observatory [11], combined with the distribution parameters from IHME GBD 2015. Population exposure to obesity and blood pressure uses data from NCD-RisC dataset [12]. For additional information on the data sources used in the model, see Table S6.

In the model, the following definition of the risk factors are used:

- Smoking refers to current smoking.
- Alcohol consumption includes the level of alcohol intake (measured in grams of pure alcohol per day) and the prevalence of binge drinking (having six (five) or more drinks per occasion in the past 12 months for men (women)).
- Obesity is defined as a body mass index above 30 kg/m^2^.
- Physical inactivity is defined as having below 600 METs. High blood pressure is defined as having a systolic blood pressure higher than 140 mmHg.

Relative risks that link RFs to diseases are determined by gender and age group. Information on relative risks was collected from IHME GBD 2016 [13], the DYNAMO-HIA model [14], and the OECD alcohol model [15].

**Table S6. Data source for the model**

| **Model module** | **Parameter** | **Source** |
| --- | --- | --- |
| Demography | Population projection | United Nations. World Population Prospects - Population Division - United Nations [dataset]. Accessed on January 2018. Available from: <https://population.un.org/wpp/> |
|  | Mortality | Human Mortality Database [dataset]. Accessed on 29 March 2016. Available from: <http://www.mortality.org/> |
| Cost | Health expenditures | For France: https://www.amse-aixmarseille.fr/sites/default/files/research/cost_calculations_france.pdf  For Estonia: <https://www.amse-aixmarseille.fr/sites/default/files/research/cost_calculations_estonia.pdf>  For the Netherlands: https://www.amse-aixmarseille.fr/sites/default/files/research/cost_calculations_netherlands.pdf |
| Diseases | Disease incidence, prevalence, remission and fatality | GBD 2016 Disease and Injury Incidence and Prevalence Collaborators, Vos T, Abajobir A, Abate K, Abbafati C, Abbas K, Abd-Allah F, et al. Global, regional, and national incidence, prevalence, and years lived with disability for 328 diseases and injuries for 195 countries, 1990-2016: a systematic analysis for the Global Burden of Disease Study 2016. The Lancet. 2017;390(10100):1211-1259. |
| Risk Factors | Smoking, physical inactivity, and salt intake | GBD 2016 Disease and Injury Incidence and Prevalence Collaborators, Vos T, Abajobir A, Abate K, Abbafati C, Abbas K, Abd-Allah F, et al. Global, regional, and national incidence, prevalence, and years lived with disability for 328 diseases and injuries for 195 countries, 1990-2016: a systematic analysis for the Global Burden of Disease Study 2016. The Lancet. 2017;390(10100):1211-1259. |
|  | Alcohol | World Health Organization, Global Health Observatory Data Repository, Average daily intake in grams of alcohol, by country [dataset]. Accessed on 21 December 2017. Available from: <http://apps.who.int/gho/data/node.main.A1037?lang=en>  GBD 2015 Risk Factors Collaborators, Forouzanfar M, Afshin A, Alexander L, Anderson H, Bhutta Z, Biryukov S, et al. Global, regional, and national comparative risk assessment of 79 behavioural, environmental and occupational, and metabolic risks or clusters of risks, 1990–2015: a systematic analysis for the Global Burden of Disease Study 2015. The Lancet. 2016;388(10053):1659-1724 |
|  | Obesity and blood pressure | NCD Risk Factor Collaboration (NCD-RisC). Worldwide trends in body-mass index, underweight, overweight, and obesity from 1975 to 2016: a pooled analysis of 2416 population-based measurement studies in 128·9 million children, adolescents, and adults. The Lancet. 2017:390(10113):2627-2642. |
|  | Relative risks | Gakidou E, Afshin A, Abajobir A, Abate K, Abbafati C, Abbas K, et al. Global, regional, and national comparative risk assessment of 84 behavioural, environmental and occupational, and metabolic risks or clusters of risks, 1990–2016: a systematic analysis for the Global Burden of Disease Study 2016. The Lancet. 2017;390(10100):1345-1422.  Lhachimi S, Nusselder W, Smit H, van Baal P, Baili P, Bennett K, et al. Dynamo-HIA-a dynamic modeling tool for generic health impact assessments. PLoS ONE. 2012;7(5): e33317.  Cecchini M, Devaux M, Sassi F. Assessing the impacts of alcohol policies: A microsimulation approach. OECD Health Working Paper No. 80, Paris. 2015. |

# References

1. Cortaredona S, Ventelou B. Cost-of-illness analysis in the EGB database. 2017. Available: https://www.amse-aixmarseille.fr/sites/default/files/research/cost_calculations_france.pdf

2. Thiébaut SP. Cost-of-Illness Analysis for Estonia. 2018. Available: https://www.amse-aixmarseille.fr/sites/default/files/research/cost_calculations_estonia.pdf

3. Thiébaut SP. Cost-of-Illness Analysis for the Netherlands. 2018. Available: https://www.amse-aixmarseille.fr/sites/default/files/research/cost_calculations_netherlands.pdf

4. Christoph AUBRECHT, Carla BLÁZQUEZ, David CANTARERO BC, Sebastien CORTAREDONA, Marion DEVAUX, Mario KÖSTL SL, Alienor LEROUGE, Audrey MICHEL-LEPAGE, Jean-Paul MOATTI A, PARAPONARIS, Marta PASCUAL, Thierry PELLEGRINI FR, Berta RIVERA, Franco SASSI, Klaus STEINNOCHER BV. Validated European Health Policy Model software and documentation: FRESHER report. 2016. Available: https://www.foresight-fresher.eu/content/uploads/2018/03/d5-1-validated-european-health-policy-model-software-and-documentation-corrected.pdf

5. Thiébaut SP, Barnay T, Ventelou B. Ageing, chronic conditions and the evolution of future drugs expenditure: A five-year micro-simulation from 2004 to 2029. Appl Econ. 2013. doi:10.1080/00036846.2011.633895

6. Norton E, Deb P, Manning WG. Health Econometrics Using Stata. College Station, TX.: Stata Press; 2017.

7. Devaux M, Lerouge A, Giuffre G, Giesecke S, Baiocco S, Ricci A, et al. How will the main risk factors contribute to the burden of non-communicable diseases under different scenarios by 2050? A modelling study. PLoS One. 2020. doi:10.1371/journal.pone.0231725

8. Cecchini M, Cortaredona S, Devaux M, Elfakir A, Goryakin Y LA et al. Scientific paper on the methodology, results and recommendation for future research, FRESHER report Deliverable 5.3. 2017. Available: https://www.foresight-fresher.eu/content/uploads/2018/03/d5-3-scientific-paper-on-the-methodology-results-and-recommendation-for-future-research.pdf

9. Devaux M, Lerouge A, Ventelou B, Goryakin Y, Feigl A, Vuik S, et al. Assessing the potential outcomes of achieving the World Health Organization global non-communicable diseases targets for risk factors by 2025: is there also an economic dividend? Public Health. 2019. doi:10.1016/j.puhe.2019.02.009

10. Vos T, Abajobir AA, Abbafati C, Abbas KM, Abate KH, Abd-Allah F, et al. Global, regional, and national incidence, prevalence, and years lived with disability for 328 diseases and injuries for 195 countries, 1990-2016: A systematic analysis for the Global Burden of Disease Study 2016. Lancet. 2017. doi:10.1016/S0140-6736(17)32154-2

11. World Health Organization. Global Health Observatory Data Repository, Indicator on Average daily intake in grams of alcohol, by country. 2017. Available: http://apps.who.int/gho/data/node.main.A1037?lang=en

12. NCD Risk Factor Collaboration. Worldwide trends in body-mass index, underweight, overweight, and obesity from 1975 to 2016: a pooled analysis of 2416 population-based measurement studies in 128·9 million children, adolescents, and adults. Lancet. 2017. doi:10.1016/S0140-6736(17)32129-3

13. Abajobir AA, Abate KH, Abbafati C, Abbas KM, Abd-Allah F, Abdulle AM, et al. Global, regional, and national comparative risk assessment of 84 behavioural, environmental and occupational, and metabolic risks or clusters of risks, 1990–2016: a systematic analysis for the Global Burden of Disease Study 2016. Lancet. 2017;390. doi:10.1016/S0140-6736(17)32366-8

14. Lhachimi SK, Nusselder WJ, Smit HA, van Baal P, Baili P, Bennett K, et al. Dynamo-HIA-a dynamic modeling tool for generic health impact assessments. PLoS One. 2012. doi:10.1371/journal.pone.0033317

15. Cecchini M, Devaux M, Sassi F. Assessing the impacts of alcohol policies: a microsimulation approach. 2015. doi:10.1787/5js1qwkvx36d-en

1. It’s important to emphasize that since the estimation is based on the medical claims data, the predicted costs will apply to people with diagnosed conditions only. [↑](#footnote-ref-1)
2. We will explain later the rationale for why we estimate the probability P(C>0), rather than P(C>0|diabetes=1). [↑](#footnote-ref-2)
3. In other words, we assume that the cost differentials we observe today will keep constant in future years as well. [↑](#footnote-ref-3)
4. <http://stats.oecd.org> [↑](#footnote-ref-4)
